# Growing a new human kidney

**DOI:** 10.1016/j.kint.2019.04.040

**Published:** 2019-10

**Authors:** Adrian S. Woolf

**Affiliations:** 1 Division of Cell Matrix Biology and Regenerative Medicine, School of Biological Sciences, Faculty of Biology Medicine and Health, University of Manchester, United Kingdom; 2 Royal Manchester Children’s Hospital, Manchester University NHS Foundation Trust, Manchester Academic Health Science Centre, Manchester, United Kingdom

**Keywords:** disease, gene, mesonephros, metanephros, organoid, regeneration, stem cell

## Abstract

There are 3 reasons to generate a new human kidney. The first is to learn more about the biology of the developing and mature organ. The second is to generate tissues with which to model congenital and acquired kidney diseases. In particular, growing human kidneys in this manner ultimately should help us understand the mechanisms of common chronic kidney diseases such as diabetic nephropathy and others featuring fibrosis, as well as nephrotoxicity. The third reason is to provide functional kidney tissues that can be used directly in regenerative medicine therapies. The second and third reasons to grow new human kidneys are especially compelling given the millions of persons worldwide whose lives depend on a functioning kidney transplant or long-term dialysis, as well as those with end-stage renal disease who die prematurely because they are unable to access these treatments. As shown in this review, the aim to create healthy human kidney tissues has been partially realized. Moreover, the technology shows promise in terms of modeling genetic disease. In contrast, barely the first steps have been taken toward modeling nongenetic chronic kidney diseases or using newly grown human kidney tissue for regenerative medicine therapies.

The idea that a human kidney might be created *de novo* in the laboratory is inspiring not only for nephrologists and renal scientists; it also provides hope for people affected by kidney disease. The notion also stimulates interest in journalists and academic press offices, and an Internet search using the phrase “human kidney grown in the laboratory” reveals hundreds of posts during the past few years. In fact, a paper showing that an anatomically complete and fully functional human kidney can be generated has yet to be published. However, much evidence indicates that the journey toward this end has been convincingly started, as detailed in this article. The field is moving fast, and this review is intended to be a platform for further discussion. It is not exhaustive in terms of references, and other reviews will be cited that address specific aspects in more detail. In this article, 10 questions are asked about this field, followed by answers that are currently available.

### Question 1: Why generate a new human kidney?

There are 3 reasons to generate a new human kidney. The first reason is to learn more about the biology of developing and mature organs.^1^ The second reason is to model congenital and acquired kidney diseases, each of which may be caused by genetic defects^2^ or attack from nephrotoxic chemicals, pathogenic microorganisms, or excessive physical forces. In particular, growing human kidneys in this manner ultimately should help us understand the mechanisms of common chronic kidney diseases such as diabetic nephropathy and others featuring fibrosis, as well as nephrotoxicity. Learning more about pathobiology should in turn inform new treatments to slow the progression of kidney diseases. The third reason to generate a new human kidney is to generate functional kidney tissues for use in regenerative medicine therapies. The second and third reasons to grow new human kidneys are especially compelling given the millions of persons worldwide whose lives depend on a functioning kidney transplant or long-term dialysis, as well as those with end-stage renal disease who die prematurely because they are unable to access these treatments.^3^ As detailed in this article, the aim to create a healthy human kidney has been partially realized. Moreover, the technology is promising for modeling genetic kidney diseases and acute kidney injury. In contrast, the first steps to model nongenetic chronic kidney diseases or to use newly made human kidney tissue as a therapy barely have been taken.

### Question 2: How has human kidney development been studied historically?

The technology informing the generation of human kidney tissue *de novo* did not arise in one step; rather, the state of the art has been informed by insights acquired through studies performed during the past 6 decades. Some of these historical studies are listed in Table 1.^4^, ^5^, ^6^, ^7^, ^8^, ^9^, ^10^, ^11^, ^12^, ^13^, ^14^, ^15^, ^16^, ^17^, ^18^, ^19^, ^20^, ^21^, ^22^, ^23^, ^24^, ^25^, ^26^, ^27^, ^28^

**Table 1:** Growing a new human kidney^a^

Authors and reference nos.	Achievements
Osathanondh and Potter^4^, ^5^, ^6^, ^7^	Microdissection studies of normal and malformed human embryonic kidneys
Keller *et al.*^8^	Counting glomeruli in human kidneys
Grobstein^9^	Organ culture of embryonic mouse kidneys
Klein *et al.*^10^	Manipulating mouse kidney development in organ culture by targeting a specific protein
Lindström *et al.*^11^, ^12^, ^13^	Morphologic and molecular comparisons of human and mouse native developing kidneys
Taguchi *et al.*^14^; Taguchi and Nishinakamura^15^	Definition of growth factors and other molecules that pattern intermediate mesoderm to form the metanephric mesenchyme or the ureteric bud
Taguchi and Nishinakamura^15^; Takasato *et al.*^16^; Lam *et al.*^17^; Morizane *et al.*^18^	Defining *in vitro* protocols to drive hPSCs to kidney precursor cells
Takasato *et al.*^19^; Morizane and Bonventre^20^; Hale *et al.*^21^; Wu *et al.*^22^	Generating 3D kidney organoids from hPSCs and detailed molecular profiling of these tissues
Bantounas *et al.*^23^; van den Berg *et al.*^24^; Homan *et al.*^25^	Enhancing glomerular vascularization and maturation of glomerular basement membrane using *in vivo* implants or perfusion in culture
Freedman *et al.*^26^; Benedetti *et al.*^27^; Czerniecki *et al.*^28^	Using hPSC-derived kidney structures to model genetic kidney disease and test drug therapies

hPSC, human pluripotent stem cell; 3D, 3-dimensional.

a Underpinning historical reports using native kidneys, as well as more recent studies using kidney tissues derived from pluripotent stem cells. The list is not exhaustive.

The first stepping stone was the anatomic description of human kidney development. There are three sets of kidneys in mammals: the pronephros, the mesonephros, and the metanephros. The mature organ is derived from the embryonic metanephros. Osathanondh and Potter,^4^, ^5^ using microdissection, described morphogenesis of the normal human metanephros from its origin at five weeks’ gestation. At this point the rudiment consists of an epithelial ureteric bud, a branch of the pronephric duct, that is growing into metanephric mesenchyme, a section of the intermediate mesoderm (Figure 1). The bud arborizes, with its first branches remodeling to form the urothelial lining of the renal pelvis and calyces. Subsequent branches differentiate into collecting ducts, and nephrons form near the tips of the ureteric tree. Nephrons form by a transdifferentiation process called mesenchymal to epithelial transition. Each mature nephron in the metanephros comprises a glomerulus, a proximal tubule, a loop of Henle, and a distal convoluted tubule; the latter fuses with the collecting duct. After ureteric tree branching stops around mid-gestation, collecting ducts elongate, with new nephrons forming along the path of each duct. In humans, no further nephrons are formed after 34 to 36 weeks’ gestation. The numbers of glomeruli in kidneys of healthy adults are surprisingly variable, between about 0.25 and 2.0 million per organ.^8^, ^29^ Median numbers are less in kidneys of adults who have essential hypertension,^8^ and there is a positive correlation of glomerular numbers with body weight at birth.^29^ Race is another factor that may influence glomerular numbers.^30^ The variability of glomerular numbers measured in adults may represent congenital differences,^31^ although aging also may lead to depletion of whole glomeruli and also of podocytes within glomerular tufts.^32^, ^33^

**Figure 1:**
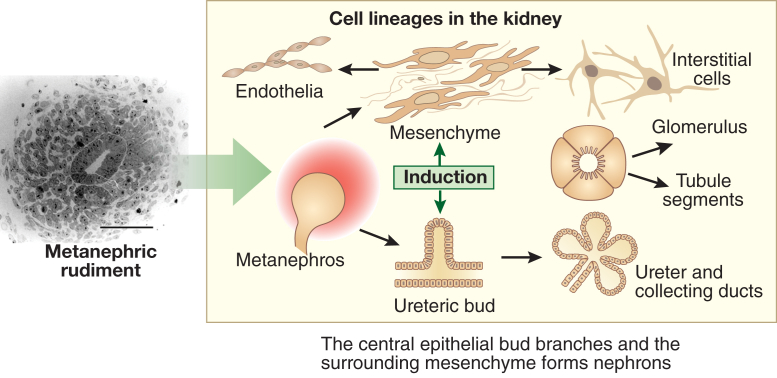
**Cell lineages in the embryonic metanephros.** The frame on the left shows the histology of the metanephros at its inception, with a central ureteric bud surrounded by metanephric mesenchyme. Bar = 50 μm. The frame on the right depicts mutual induction between these compartments. The ureteric bud differentiates into the urothelial stalk of the ureter and the arborizing collecting ducts within the kidney. The metanephric mesenchyme undergoes mesenchymal to epithelial transition to form nephrons, comprising glomerular and tubule epithelia, whereas other cells in the mesenchymal compartment will form interstitial cells and endothelia. To optimize viewing of this image, please see the online version of this article at www.kidney-international.org.

The second stepping stone was developmental biology experiments in mice that illuminated mechanisms of development. Grobstein^9^ initiated and subsequent pioneers^34^, ^35^, ^36^ optimized organ culture where embryonic kidney rudiments were maintained *ex vivo*, typically for up to a week. They demonstrated that ureteric bud branching and mesenchymal to epithelial transition proceeded as *in vivo* after whole rudiments were explanted but that the bud or metanephric mesenchyme neither differentiated nor survived when either tissue was explanted alone. This finding led to the hypothesis that each component secreted molecules that stimulated its neighbor. This idea was supported by descriptions of abnormal metanephric differentiation in mutant mice lacking specific secreted molecules, such as growth factors, and the fact that *ex vivo* manipulation of growth factor signaling modulated branching and nephron formation in organ culture.^37^, ^38^ Expression of these effector molecules, which include extracellular matrix proteins, is regulated by transcription factors. A key observation was that the embryonic mouse metanephros could be disaggregated into single cells that, after being plated as a group onto an organ culture platform, could regroup and then differentiate into rudimentary nephrons and collecting ducts.^39^, ^40^ As reviewed,^41^ mouse experiments also have helped define molecules that pattern sets of cells within the intermediate mesoderm and stimulate them to differentiate into the metanephric mesenchyme or ureteric bud lineages.^14^, ^15^ These insights are critical because, in the developing embryo, the metanephros does not arise *de novo* but itself derives from specific fields of precursor mesodermal cells. Although human fetal kidneys can be explanted and maintained in organ culture,^42^ few such studies have been performed, limited by the sparsity of precisely staged rudiments available for study.

The structure of nephrons and collecting ducts and gene expression patterns in the developing metanephros are generally similar in mice and humans.^11^, ^12^, ^13^ Furthermore, mutations of orthologues of genes directing mouse metanephric development have been reported in some people with malformed kidneys,^43^ and these genes are expressed in the human-developing metanephros.^44^, ^45^, ^46^ Clearly, however, there are certain gross differences between mouse and human kidneys, with the former containing only around 10,000 glomeruli^47^ and having one rather than multiple papillae. Moreover, metanephric nephrons are generated over two weeks in mice but over 30 weeks in humans. Intriguingly, rare but important genomic differences exist between the two species. The human gene called *KAL*, also known as *Anosmin 1*, encodes a protein coating the ureteric bud tree,^48^ and *KAL* mutations cause X-linked Kallmann syndrome, which features kidney agenesis.^49^ In mice, however, there is no convincing ortholog of *KAL*.^50^ Recent studies also reveal that some human nephron precursor cells co-express *Sine oculis homeobox homolog 1* and *Forkhead Box D1,*^12^, ^51^ whereas in mice these transcription factors are respectively confined to nephron precursors and interstitial stromal cells.^12^ Some of these differences may partly explain why the range of kidney malformation phenotypes in mice do not exactly represent those found in humans. Examples are the massive prenatal overgrowth followed by spontaneous involution, as well as metaplastic cartilage and smooth muscle transdifferentiation, which are characteristic of human multicystic dysplastic kidneys.^44^, ^52^

### Question 3: How are human pluripotent stem cells turned into kidney precursor cells?

The third line of study that informed the possibility of growing a new human kidney is pluripotent stem cell (PSC) technology. Figure 2 provides an overview of how human PSCs are generated and then turned into new kidney tissues using the various strategies described in this review. Table 1 lists some of the key studies in this field. The inner cell mass of the preimplantation embryo contains embryonic stem cells (ESCs) that can naturally form all organs and tissues found in the mature animal.^53^ ESCs also have the ability for unlimited self-renewal, making them one variety of PSCs.^53^ ESCs are isolated from early embryos; such research currently has ethical permission in only some countries and centers. More recently, it has been possible to generate “induced” PSCs (iPSCs) from adult tissues, starting from skin fibroblasts, blood cells, or even mature kidney cells.^54^ Here, the differentiated cells are programmed by forced expression of a suite of transcription factors to dedifferentiate to an ESC-like state when they become spontaneously self-renewing.^55^, ^56^, ^57^, ^58^ After being implanted into immunocompromised mice, ESCs typically form teratomas (tumors) that contain cells from the three germ layers: ectoderm (e.g., neuroepithelium), endoderm (e.g., gut epithelia), and mesoderm (e.g., cartilage and muscle; Figure 3).^23^, ^56^ Such ESC-derived teratomas can contain structures resembling the ureteric bud and pronephric duct.^59^ The utility of this technique for kidney research is limited because the renal cells are just a small subset of the total population.

**Figure 2:**
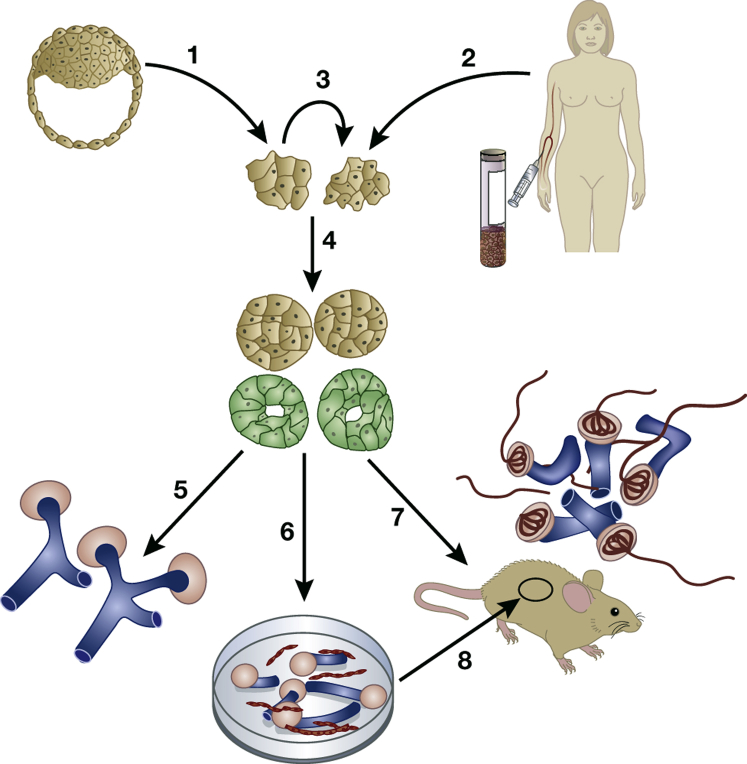
**Scheme showing how pluripotent stem cell (PSC) technology can be used to generate new kidney tissues.** Human PSCs can be generated directly from early human embryos (arrow 1) or by generating induced PSCs from mature blood, skin, or urine cells (arrow 2). Once generated, PSCs can be maintained so they undergo self-renewal (arrow 3), or they can be induced to differentiate in culture (arrow 4) into intermediate mesoderm-like cells that then begin to express molecules (green) found in the developing kidney. These kidney precursor cells can be maintained in 2-dimensional culture (arrow 5) where they form sometimes branching tubule-like structures (blue) and primitive nephrons (pink) over a few weeks. Alternatively, the PSC-derived kidney precursor cells can be dissociated and plated in 3-dimensional masses (arrow 6) that differentiate to form tubules (blue) and avascular glomeruli (pink). These organoids contain endothelia (red) between tubules. A third option is to implant the kidney precursor cells into immune-deficient mice (arrow 7), where the human cells survive subcutaneously at least for several months and form vascularized glomeruli (red inside pink structures) perfused with blood. These glomeruli contain more mature glomerular basement membranes than glomeruli that differentiate in culture. A similar result can be obtained after implanting PSC-derived kidney organoids under the kidney capsule inside immune-deficient mice (arrow 8).

**Figure 3:**
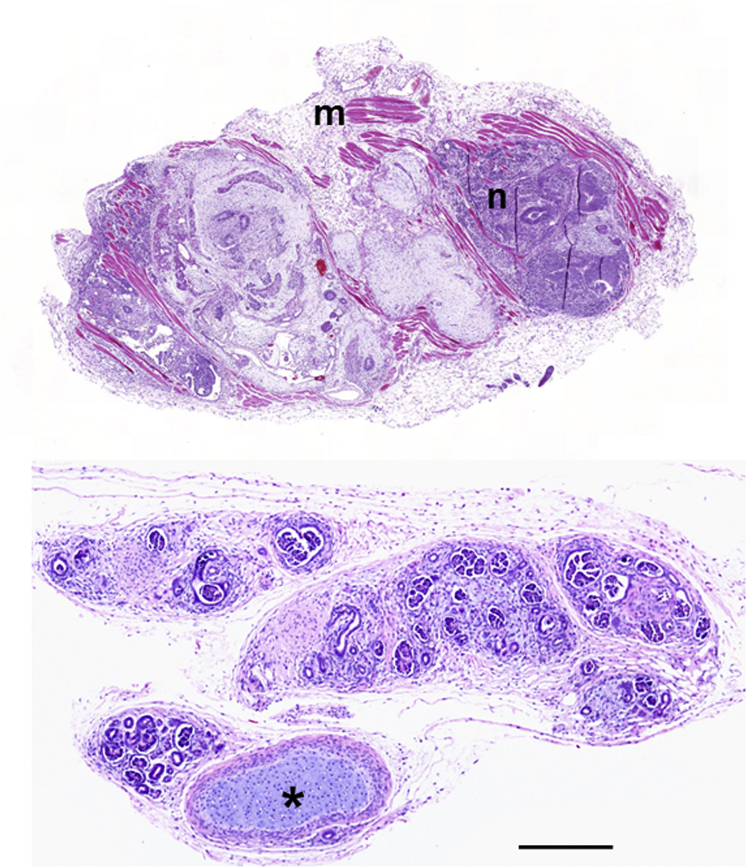
**A teratoma and a mini kidney formed *in vivo*.** The upper frame shows the histology of a teratoma, a type of tumor, that grew after implanting undifferentiated human pluripotent stem cells (hPSCs) under the skin of an immunocompromised mouse. Note muscle (m) and neural tissue (n). The lower frame shows an example of a mini kidney that formed after subcutaneous implantation of hPSC-derived kidney precursors. Note that the teratoma contains a mixture of tissues, whereas the mini kidney contains nephron-like structures, with some off-target cartilage (asterisk). Bar = 0.2 mm. Images are adapted from Bantounas I, Ranjzad P, Tengku F, et al. Generation of functioning nephrons by implanting human pluripotent stem cell-derived kidney progenitors. *Stem Cell Reports.* 2018;10:766–779^23^ via Creative Commons Attribution License (CC BY). To optimize viewing of this image, please see the online version of this article at www.kidney-international.org.

Several groups published breakthrough studies that described protocols driving human PSCs into kidney cells.^14^, ^16^, ^17^, ^18^ For example, in one protocol,^16^ wingless/integrated signaling is first upregulated by exposing stem cells to CHIR 99021, a GSK3β inhibitor, followed by exposure to fibroblast growth factor 9. Longer exposure to CHIR 99021 encourages generation of cells expressing renal mesenchymal lineage transcripts, whereas shorter exposure favors cells expressing ureteric bud markers.^19^ Adding exogenous retinoic acid shifts differentiation toward the bud lineage, whereas retinoic acid signaling blockade favors renal mesenchyme.^19^ In mice *in vivo*, the ureteric bud and renal mesenchyme arise from different sections—anterior and posterior, respectively—of the intermediate mesoderm.^14^, ^15^ Most likely, modulating wingless/integrated and retinoic acid signaling in human PSC culture drives them to become either anterior or posterior intermediate mesoderm-like cells.

In contrast to PSCs, mesenchymal stem cells (MSCs), found in bone marrow and adipose tissues, typically differentiate into cartilage and fat. In part, their apparently limited differentiation repertoire may reflect our lack of understanding as to how to unravel their full potential. Indeed, authors of one study derived kidney precursor cells from human MSCs after engineering them to express glial cell line–derived neurotrophic factor, a ureteric bud stimulating factor, and implanting them inside intact mouse embryos around the location where the metanephros normally forms.^60^

### Question 4: Are kidney organoids that have been made to date the same as native developing kidneys?

When the aforementioned human PSC protocols are used in 2-dimensional cultures, differentiation over several weeks is typically limited to formation of clusters of cells that mimic renal mesenchymal cell aggregation and mesenchymal to epithelial transition, the first steps of nephron differentiation.^16^, ^18^ However, if the PSC-derived kidney precursor cells are disaggregated and placed as a mass in organ culture, more impressive morphogenesis proceeds in 3 dimensions over a similar period. The creation of these human kidney “organoids” is a technical tour de force, but what precisely are the types of cells and structures found in these tissues? The answer depends on the exact protocol being used. Consider, for example, one pioneering protocol^19^ that has been used to differentiate numerous wild-type PCS lines in several laboratories.^21^, ^22^, ^22^, ^24^

The organoids contain glomerulus-like structures, each composed of a central cluster of cells surrounded by a space and a capsule (Figure 4^23^). The tufts contain podocyte-like cells that immunostain for Wilms tumor 1, synaptopodin, podocalyxin, laminin B2, and pan-collagen (IV). Closer inspection reveals that these structures differ from mature glomeruli found in native mature kidneys. Tuft cells in cultured human organoids do not make mature glomerular basement membrane (GBM) proteins such as collagen α3 and α4 (IV).^21^, ^23^ Moreover, the tufts rarely contain capillary loops, even though endothelial cells are detected around glomeruli.^23^ Moreover, podocytes in human (h)PSC-derived kidney organoids do not immunostain intensely for vascular endothelial growth factor A, a critical molecule for invasion of endothelia into glomeruli during normal nephrogenesis.^61^

**Figure 4:**
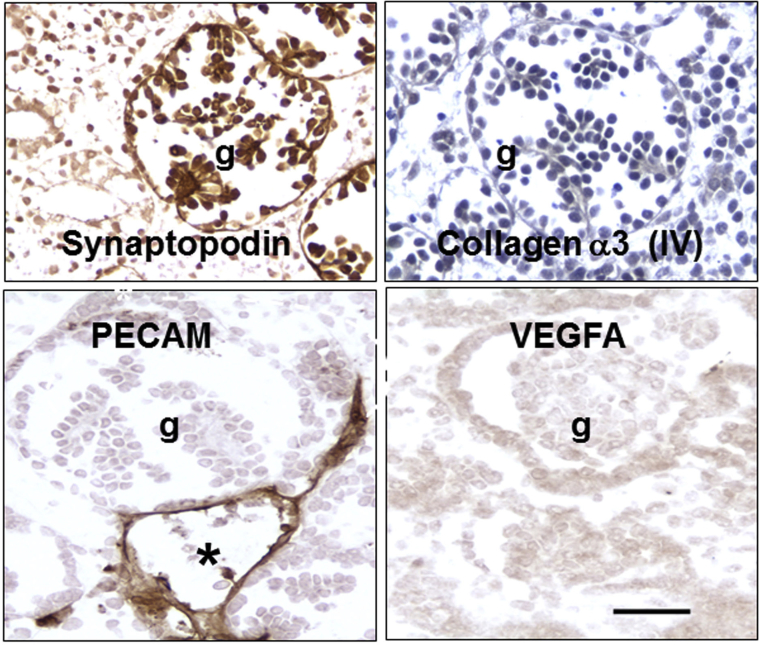
**Glomeruli formed in cultured human pluripotent stem cell–derived human organoids are immature.** Glomerular tufts (g) immunostain (brown) for the podocyte marker synaptopodin. Glomeruli do not immunostain for collagen α3 (IV) or vascular endothelial growth factor A (VEGFA). Although capillaries that immunostain for platelet endothelial cell adhesion molecule (PECAM) are detected around the glomerulus (the asterisk indicates the lumen of one such vessel), they are rarely found inside the tuft. Nuclei in the collagen α3 (IV) frame are counterstained with hematoxylin. Bar = 50 μm. Images are adapted from Bantounas I, Ranjzad P, Tengku F, et al. Generation of functioning nephrons by implanting human pluripotent stem cell-derived kidney progenitors. *Stem Cell Reports.* 2018;10:766–779^23^ via Creative Commons Attribution License (CC BY). To optimize viewing of this image, please see the online version of this article at www.kidney-international.org.

Given classic organ culture studies of murine metanephric kidneys, these defects should not come as a surprise. Glomeruli formed in this context are also avascular, and they lack mesangial cells.^62^ Murine metanephric explants contain endothelial cells that can be stimulated to proliferate by low oxygen tensions, but they predominantly remain in the interstitium.^63^, ^64^ In normal murine development, GBM undergoes molecular maturation, with laminin trimers changing from LM-111 to LM-521, each made by both podocytes and endothelia.^65^ The immature GBM contains collagen α1α2α1(IV), again made by glomerular endothelia and podocytes, whereas collagen α3α4α5(IV) in the mature GBM is solely derived from podocytes. In organ culture, even though podocytes are present, they do not seem to be able to generate their mature repertoire of mature proteins, most likely because this process is somehow stimulated by the proximity of endothelia and perhaps also by mechanical forces conferred by blood flow.

The same organoids contain tubules, some of which structurally resemble proximal and distal tubule nephron segments.^19^, ^23^ These tubules immunostain for proteins found in normal proximal (e.g., aquaporin-1 and cubulin) or distal (e.g., TRPV5) tubules. Whereas some tubules immunostain for proteins expressed by collecting ducts, such as paired box 2, GATA binding protein 3, and cadherin 1, transcriptomes of single cells show that they do not express the full repertoire of RNAs characterizing collecting duct cells *in vivo*.^22^ There are two possible interpretations here, and presently the jury is still undecided. Either some of these tubules are collecting ducts that have not fully matured or they are composed of quite different cells, perhaps more closely resembling distal tubules of the nephron. Similar phenotyping observations have been made for kidney tissues generated using another hPSC protocol.^18^, ^20^ A third 3-dimensional (3D) protocol, which first drives human PSCs into ureteric bud precursors, has generated branched structures^15^ more closely resembling the first morphogenetic steps of ureteric tree arborization described in the native human metanephros.^5^, ^8^ The normal kidney is divided into the cortex, outer medulla, and inner medulla. By contrast, human kidney organoids show little evidence of such patterning. In the future, adapting culture techniques, as have been applied to murine metanephric organ cultures,^40^ may improve tissue layering of human organoids.

Importantly, at least at first glance, kidney organoids can contain some surprising cell types, as assessed by gene expression profiling and antibody staining. For example, neural-like cells can compose up to 10% of the whole kidney organoid population, and certain cells express neural growth factors or their receptors.^22^ Although this may represent an “off target” neuronal result, embryonic rodent metanephric kidneys contain spindle-shaped stromal cells that express neurofilament proteins.^66^ Moreover, developing native human and rodent kidneys express a variety of nerve growth factors,^67^, ^68^ although it is important to note that nonneural kidney cells can express nerve growth factor receptors.^68^ Classic developmental biology studies showed that renal mesenchyme was stimulated to undergo mesenchymal to epithelial transition when placed near embryonic spinal cord neural cells.^69^ *In vivo*, however, neurons are not detected in the embryonic metanephros until after mesenchyme has been induced to begin to differentiate.^69^ hPSC-derived kidney organoids also contain smooth muscle-like cells between tubules, and the proportion of the myofibroblasts appears to increase with prolonged culture.^70^

### Question 5: Do human kidney organoids more closely resemble the metanephros or the mesonephros?

One might conclude that human kidney organoids generated and maintained in 3D culture are ersatz kidneys, poor imitations that are neither fully mature nor containing the right types of cells. A related question is whether they might even be the “wrong type” of embryonic kidney. Although the mature mammalian kidney forms from the embryonic metanephros, in the first 10 weeks of human gestation it is the mesonephric kidney that is the larger organ. Moreover, both the mesonephros and metanephros arise from intermediate mesoderm.^1^ Unlike the mouse mesonephros that contains tubules but no formed glomeruli,^71^ the human mesonephros *in vivo* contains nephrons, with each comprising a prominent vascularized glomerulus, a proximal-like tubule, and a short distal-like tubule, with the latter connected to the pronephic duct by a short collecting duct-like segment.^72^, ^73^, ^74^ Based on morphology alone, human kidney organoids generated from PSCs by certain protocols^18^, ^19^ could indeed represent human mesonephric or immature metanephric kidneys. Human mesonephric kidneys also lack loops of Henle, and serial branching does not occur. Notably, human kidney organoids tend to have only rudimentary loops of Henle, and branching is limited.^18^, ^19^ The normal *in vivo* fate of the human mesonephros is to degenerate over several months *in utero*, whereas the metanephros will survive, grow, and mature. In the future, studies are needed to comprehensively compare gene expression patterns between human mesonephric and metanephric kidneys. Current knowledge about these gene expression patterns is limited,^75^ and perhaps there will be profiles that distinguish these two embryonic organs.

### Question 6: What happens when hPSC-derived kidney precursors are placed into a living animal?

If differentiation is somewhat limited in 2-dimensional and 3D cultures, can the situation be improved by placing the kidney precursors in a more lifelike environment? One group^23^ dissociated hPSC-derived kidney precursor cells, embedded them in an extracellular matrix-like material, and then implanted several hundred thousand cells under the skin of immunodeficient mice (Figure 2). After three months, viable implants were visualized in living mice, as assessed by imaging bioluminescence generated by virtue of the cells having been transduced with a *luciferin* reporter gene. Histology revealed that the implants contained a similar range of cells as found in cultured organoids, but the glomeruli were more mature. Not only did glomeruli contain capillary loops, with at least some endothelia of human origin, but they also contained mesangial-like cells. As assessed by electron microscopy, sections of the GBM appeared to have a normal fetal trilaminar ultrastructure, and electron-dense areas were found between foot processes, recalling slit diaphragms in mature glomeruli. Furthermore, in contrast to podocytes in cultured organoids, those formed in implants immunostained prominently for vascular endothelial growth factor A and collagen α3 (IV) (Figure 5^23^). Another research group harvested human organoids that had begun to undergo morphogenesis in culture and implanted them under the kidney capsule of immunodeficient mice.^24^ Again, the glomeruli that formed contained capillary loops, and it was demonstrated that these loops were perfused, as assessed by visualizing the appearance of a fluorescent molecule injected into the host vasculature.

**Figure 5:**
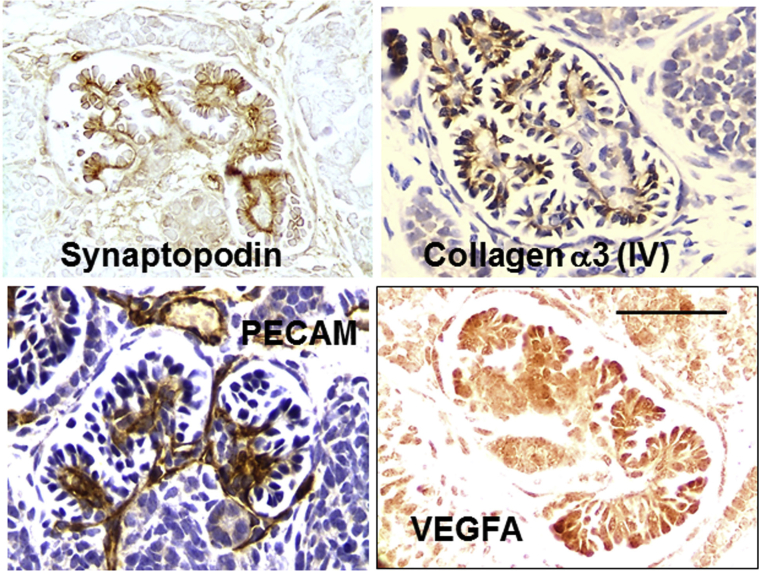
**Mature glomeruli formed *in vivo* after implanting human pluripotent stem cell–derived kidney precursor cells.** Glomerular tufts immunostain (brown) for the podocyte marker synaptopodin. Glomeruli immunostain for collagen α3 (IV) and vascular endothelial growth factor A (VEGFA). Capillaries that immunostain for platelet endothelial cell adhesion molecule (PECAM) are detected inside the glomerular tuft. Nuclei in the collagen α3 (IV) and PECAM frames are counterstained with hematoxylin. Bar = 50 μm. Images are adapted from Bantounas I, Ranjzad P, Tengku F, et al. Generation of functioning nephrons by implanting human pluripotent stem cell-derived kidney progenitors. *Stem Cell Reports.* 2018;10:766–779^23^ via Creative Commons Attribution License (CC BY). To optimize viewing of this image, please see the online version of this article at www.kidney-international.org.

The aforementioned results are consistent with long-standing observations from murine developing biology studies that show native kidney rudiments can develop after transplantation.^76^, ^77^, ^78^, ^79^, ^80^ In fact, similar transplant experiments have been reported using native human metanephric kidney donor tissue.^81^ Mouse embryonic metanephric kidneys contain a subset of cells that express endothelial lineage molecules, such as vascular endothelial growth factor and angiopoietin receptors,^82^, ^83^ and these contribute to glomerular capillaries when rudiments are implanted, for example, into the cortex of host kidneys.^83^ The *in vivo* scenario most likely also allows the ingrowth of small host blood vessels that contribute to capillaries in the “mini kidney.” Perhaps, in the future, the richness of a kidney organoid blood supply could be increased by implanting kidney precursor cells in vascularized chambers, as has been done with other types of precursor cells.^84^

### Question 7: What further steps are needed to generate a functioning new human kidney and urinary tract?

At the time of writing, the “state of the art” is the generation of human kidney organoids that contain vascularized glomeruli, structures resembling proximal and distal tubules, and perhaps also collecting ducts. However, we are still far from a realistic “kidney.’” First there is a problem of scale, with each organoid formed in culture or after implantation^23^ containing perhaps several tens of nephrons and being at the most about 1 cm across (Figure 6^23^). When compared with the glomerular number and dimension of a mature human kidney, the organoid is several orders of magnitude smaller. Such implants of hPSC-derived kidney precursor cells contain glomeruli with mature-looking GBM and perfused capillaries, so they might be able to filter blood. After i.v. injection of fluorescent low molecular weight dextran into the mouse host, a subset of tubules in tissues derived from implanted hPSC-derived kidney precursors contain the fluorescence, consistent with at least a low level of ultrafiltration.^23^ Indeed, this outcome is not surprising because a degree of glomerular ultrafiltration has been documented after transplanting native murine and human metanephric kidneys.^77^, ^80^, ^81^ Challenges remain, however; the first is the small number of nephrons per implant, and the second is that implants lack high pressure and volume renal arteries, instead being fed by small vessels. Importantly, the microvasculature within mature native kidneys does not simply comprise glomerular capillaries; there are also postglomerular capillary beds both in the cortex that receive fluids reabsorbed by proximal tubules and vasa rectae in the outer medulla. These capillary beds, as well as arteries, veins, and lymphatics, would need to be generated in the realistic *de novo* kidney. In the future, the potential functionality of kidney tubules formed from hPSCs will need to be studied in detail, and most likely this will require physiologic analyses of vascularized organoids *in vivo* or in organoids maintained in culture.

**Figure 6:**
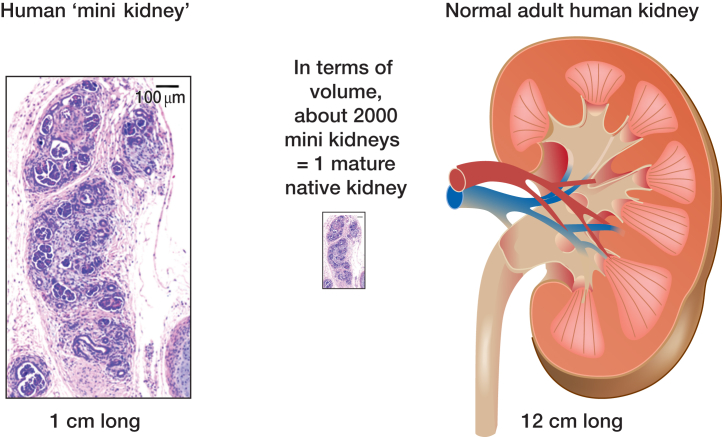
**Comparison of a human pluripotent stem cell (PSC)–derived kidney organoid with a native mature kidney.** Note the PSC-derived mini kidney is only 1 cm long. Around 2000 of these would constitute a similar volume as a native adult human kidney. Note also that the organoid lacks a renal artery and vein and it has no renal pelvis or ureter, all of which are present in the mature native kidney. The image on the left is adapted from Bantounas I, Ranjzad P, Tengku F, et al. Generation of functioning nephrons by implanting human pluripotent stem cell-derived kidney progenitors. *Stem Cell Reports.* 2018;10:766–779^23^ via Creative Commons Attribution License (CC BY). To optimize viewing of this image, please see the online version of this article at www.kidney-international.org.

The third major challenge here is that kidney organoids lack a renal pelvis and a ureter, so any nascent urine generated would simply diffuse back into host tissues. One solution would be to use surgery to fashion a urinary conduit from embryonic ureter and/or bladder tissues. Indeed, such strategies have been used to plumb kidneys grown from native metanephric rudiments.^80^, ^85^ An alternative would be to generate the lower urinary tract from human stem cells. Although this procedure currently is not possible, first steps have been taken toward this end. As for the metanephros, mouse developmental biology studies point to molecules that direct the development of the ureter and urinary bladder.^37^, ^86^, ^87^, ^88^, ^82^ Furthermore, studies of native human bladder cells have explored their differentiation in tissue culture.^89^ There are several reports of programming of PSCs to cells that express smooth muscle and urothelial markers,^90^, ^91^, ^92^ while MSCs can be induced to differentiate into bladder smooth muscle–like cells.^93^ However, the state of the art appears to be some way from growing a new lower urinary tract from stem cells.

### Question 8: Can new human kidneys serve as models for understanding disease?

The experimental *de novo* generation of human kidney diseases would constitute a breakthrough in renal research. In theory, human kidneys grown *de novo* might be more realistic than either kidneys in experimental animal or conventional cell lines, which tend to lose their differentiated phenotypes in culture. As previously described, the kidneys that currently can be generated from hPSCs have the advantages of being 3D structures that contain a variety of renal tissues. In organoid culture these structures take several weeks to form, and even then they are not fully mature because they lack, for example, mature GBMs and glomerular capillaries. Moreover, there is doubt about whether such organoids can be maintained in culture for longer periods without showing a loss of viability and a degree of transdifferentiation to, for example, myofibroblasts.^70^

With the aforementioned caveats, kidney organoids have been utilized in experiments in which chemicals were used to damage podocytes or tubules.^21^, ^26^ Let us now consider whether kidney organoids might be suitable for modeling chronic kidney diseases. One of the most common chronic kidney diseases is diabetic nephropathy. Here, high concentrations of glucose and other metabolic disturbances perturb the kidney over many months or years. The facts that kidney organoids can be maintained in culture only for a matter of weeks and their lack of perfused mature glomeruli *a priori* limit their potential to model diabetic nephropathy. Perhaps it would be more logical to model diabetic nephropathy in mini kidneys generated after implanting PSC-derived precursors. Here, at least, the tissues contain mature glomeruli perfused with blood. However, there is another problem with these approaches: the first weeks in organ culture mirror a developing kidney, where precursor cells are still differentiating. Therefore, investigators who perform experiments using exposure to high concentrations of glucose actually will be studying potential teratogenic effects on organogenesis rather than modeling diabetic nephropathy in mature organs. These caveats are important given the strong human clinical and animal experimental evidence that maternal diabetes affects kidney development.^94^, ^95^, ^96^ Similar drawbacks would apply to using human kidney organoids to model chronic renal scarring due to, for example, transforming growth factor–β.^97^ Exposure of kidney organoids to this molecule would be modeling its effects on retarding nephrogenesis and causing metaplasia,^98^, ^99^ as much as modeling chronic interstitial fibrosis. On the other hand, such manipulations may be excellent ways of studying effects of these agents on human kidney development, for instance, modeling the phenotypes of dysplastic kidneys.^6^, ^7^, ^100^, ^101^

First steps have been made to model genetic kidney diseases using human kidney organoids. Again it could be argued that current models, which feature structures that are still maturing, would be more suited for reproducing congenital malformations rather than genetic diseases where kidneys show disease phenotypes in more mature structures. Studies have been derived from iPSC affected patients, or mutated specific genes in wild-type PSCs using editing with clustered regularly interspaced short palindromic repeats technology.^102^, ^103^ Using these strategies, investigators have begun to study the effects: on kidney precursors conferred by mutations of *Hepatocyte nuclear factor-1B* and *Paired box 2*; on podocytes conferred by mutations of *Podocalyxin*, *Nephrin,* and *Podocin*; and on tubules conferred by mutations of *Polycystic kidney disease 1* or *2*, *Inositol polyphosphate 5-phosphatase*, and *Intraflagellar transport 140*.^21^, ^26^, ^27^, ^28^, ^104^, ^105^, ^106^, ^107^ The ability to generate kidney cysts from mutated PSCs, for example, is beginning to be used as a test bed to identify cyst-blocking drugs.^28^

### Question 9: Are there informative strategies other than growing hPSC-derived kidney organoids?

Generating new kidney cells on physical substrates offers the promise of a “lifelike” environment in cell culture. One such approach is to differentiate hPSCs into mature podocytes in “microfluidic glomerulus chip” that mimics blood flow and allows ultrafiltration in a 3D environment.^108^, ^109^ This approach has been used to recreate healthy glomeruli, as well as those damaged by a nephrotoxin.^108^, ^109^ The technique also offers promise to study the effects of altered physical forces on glomeruli—for example, modeling glomerular damage from hypertension.^110^ In theory, a whole nephron could be built up using precursor cell and chip technology, for example, with a perfused glomerulus in series with a proximal tubule.^111^, ^112^ Another technique is to use PSCs to repopulate extracellular matrix scaffolds composed of kidneys enzymatically depleted of native cells.^113^, ^114^, ^115^ An attraction here is that such scaffolds can be cryopreserved for several months before use.^115^ Recently, chip-based technology has been applied to PSC-derived human kidney organoids to show that tissues perfused with media have an expanded vascular compartment and more mature glomeruli.^25^

An alternative technology to generating kidney tissues from PSCs, whether ESCs or iPSCs, is to used forced expression of nephrogenic transcription factors in adult renal or nonrenal cells to reprogram them to a specifically kidney precursor cell state; from this point they can be induced to differentiate into more mature kidney cells.^116^, ^117^ Finally, although beyond the remit of this review that considers the experimental *de novo* creation of kidneys, it is notable that populations of precursor-like cells can be harvested from native fetal or mature kidneys and stimulated to differentiate into nephrons.^118^, ^119^, ^120^, ^121^

### Question 10. Can new human kidneys be used as therapies?

In nonrenal diseases, precursor cell technology is being applied to generate new tissues for regenerative medicine therapies. For example, human epidermal stem cells can be isolated and a therapeutic gene introduced, after which they are expanded to form a new skin to treat junctional epidermolysis bullosa.^122^ Human ESCs can be differentiated into retinal pigment cells and used to ameliorate macular degeneration.^123^ iPSCs induced to differentiate into dopaminergic neurons have been used to treat nonhuman primates with Parkinson disease,^124^ in anticipation of a human trial.

MSCs and renal precursor cells have been administered in murine models of acute or chronic nephropathy, sometimes ameliorating aspects of the disease.^125^ In these cases, the basis for efficacy is unlikely to be explained by directly conferring functional kidney tissue derived from administered cells; instead, effects are more likely to be explained by immunomodulatory, paracrine, or microvesicle-mediated mechanisms.^125^, ^126^, ^127^ The long-term fates of such administered cells is currently uncertain. Although a minority may even integrate with host kidney epithelia,^128^ most i.v. injected precursor cells are sequestered in the lung, and cells injected into the renal artery may embolize rather than get to the parenchyma.^114^, ^129^

Another study found that, after undifferentiated hPSCs were implanted into remnant rat kidneys, they formed proliferative masses that resembled Wilms tumor, a childhood kidney cancer.^130^ Although iPSC-derived human kidney precursor cell populations should be devoid of stem cells, it is of potential concern that implanted hPSC-derived kidney precursors might form cancers. To date, the fates of such implanted cells have been limited to inspections up to a few weeks to months.^23^, ^24^, ^130^ At this point, along with glomeruli and tubules, implants contain zones with undifferentiated mesenchymal-like cells and “off-target” cartilage-like cells. Although cells appear confined to their implanted site, as assessed by live imaging of a reporter gene,^23^ exhaustive autopsy studies have yet to be performed seeking human cells in other sites. In the future, it will be informative to undertake detailed gene expression profiles to help unravel whether cultured organoids, or tissues formed after implantation of hPSC-derived kidney precursors, have Wilms tumor–like characteristics.^131^, ^132^, ^133^, ^134^

Human trials that use MSCs to treat kidney disease are under way. In one trial, MSCs were seeded into hollow-fiber hemofilters with the aim “to reprogram the molecular and cellular components of blood” of people with acute kidney injury.^135^ In another preliminary safety trial, MSCs were infused after conventional kidney transplantation in the hope that their antiinflammatory and immune-regulatory properties will benefit graft outcomes.^136^ Careful follow-up is required in such human studies because experiments show that mouse MSCs can form tumors after being administered to mice and that this outcome is dependent on the route of administration and the strain of the host. Moreover, certain human MSCs can proliferate after i.v. administration into immunodeficient mice.^129^

As yet, newly grown human kidney organoids have not been used as renal replacement therapy, either in preclinical models or clinical trials. Apart from potential tumorgenicity, as detailed earlier, there are still problems to overcome involving the small scale of organoids versus a native kidney and the facts that they lack a robust renal vasculature and urinary drainage system.

### Conclusions

The aim to create healthy human kidney tissues has been partially realized. Moreover, the technology shows promise in terms of modeling genetic disease. By contrast, barely the first steps have been taken toward modeling nongenetic chronic kidney diseases or using newly grown human kidney tissue for regenerative medicine therapies.

## Disclosure

The author declared no competing interests.
